# A Case Series of Pediatric Intestinal Ganglioneuromatosis With Novel Phenotypic and Genotypic Profile

**DOI:** 10.3389/fmed.2022.883958

**Published:** 2022-06-16

**Authors:** Yuan Fang, Ye Zhang, Rui Dong, Yi-zhen Wang, Lian Chen, Gong Chen

**Affiliations:** ^1^Department of Pathology, Anhui Provincial Children’s Hospital, Hefei, China; ^2^Department of Gastroenterology, Children’s Hospital of Fudan University, Shanghai, China; ^3^Department of Oncological Surgery, Children’s Hospital of Fudan University, Shanghai, China; ^4^Department of Pathology, Children’s Hospital of Fudan University, Shanghai, China; ^5^Department of Pediatric Surgery, Children’s Hospital of Fudan University, Shanghai, China

**Keywords:** intestinal ganglioneuromatosis, juvenile polyps, pseudomembranous enteritis, mutation, case series

## Abstract

**Introduction:**

Intestinal ganglioneuromatosis (IGN) is a rare condition with enteric involvement. Herein, we report a case series of pediatric IGN with a novel phenotypic and genotypic profile.

**Methods:**

The clinical presentation, histopathology, immunochemistry, molecular features, treatment, and prognosis of 3 cases of IGN were assessed.

**Results:**

The cases involved 3 boys with an age range of 1 year and 4 months to 8 years, mimicking juvenile polyps or pseudomembranous enteritis. One patient carried a novel germline mutation in *RTEL1* (c.296C > T/p.Pro99Leu) along with variants in *F11* (c.1489C > T/p.Arg497Xaa), *NBAS* (c.1514delC/p.Pro505Hisfs*15), and *FECH* (c.315-48T > C/splicing), who died due to intractable inflammation. The other two patients underwent recurrence without significant signs of systemic syndrome or malignant progression.

**Conclusion:**

This case series added to the phenotypic and genotypic spectrum of pediatric IGN, which requires the accumulation of more cases and research for in-depth understanding.

## Introduction

Ganglioneuroma (GN) is a well-differentiated benign tumor involving a combination of neuroblastoma (NB) and ganglioneuroblastoma (GNB) to compose a neuroblastic tumor (NT) according to the International Risk Group (INRG) (1). GN originates from the primitive neural crest, mostly the adrenal gland, followed by the retroperitoneum, mediastinum, neck, and pelvic sympathetic ganglia (2). In addition to a rare condition with enteric involvement, intestinal ganglioneuromatosis (IGN) is characterized by the proliferation of ganglion cells, Schwann stromal cells, and nerve fibers in the lamina propria, submucosa, and/or myenteric plexus of the intestinal wall. The disease can be divided into polypoid ganglioneuroma, ganglioneuromatous polyposis, and diffuse ganglioneuromatosis, depending on the number of lesions and growth pattern (3). The latter is usually associated with systemic disorders, including multiple endocrine neoplasia type 2B (MEN2B), neurofibromatosis type 1 (NF1), and Cowdom syndrome (4–6). Clinically, IGN often mimics Crohn’s disease (7) or gastrointestinal stromal tumor (GIST) (8). Death due to delayed diagnosis in a 6-year-old boy has been reported (9), rendering IGN a crucial threat to the health of children.

Herein, we reported 3 pediatric cases of IGN manifesting as recurrent bloody stools or watery diarrhea, with a preliminary diagnosis of juvenile polyps or pseudomembranous enteritis. The cases were finally diagnosed as IGN based on the proliferation of ganglion cells, Schwann stromal cells, and nerve fibers in the lamina propria, submucosa, and/or myenteric plexus by repeated biopsy or full-thickness resection. Whole-exome sequencing (WES) revealed a novel germline mutation in *RTEL1* (c.296C > T/p.Pro99Leu), along with the potentially pathogenic variants in *F11* (c.1489C > T/p.Arg497Xaa), *NBAS* (c.1514delC/p.Pro505Hisfs*15), and *FECH* (c.315-48T > C/splicing) in one patient, who died owing to intractable inflammation. The other two patients underwent recurrent polypectomy without significant signs of systemic syndrome or malignant progression. This work may enhance practitioners’ awareness and arouse further research.

This article is written in accordance with CAse REport (CARE) Checklist (10), which is uploaded as Supplementary Material.

## Materials and Methods

### Patients

The clinical data, histopathology, immunohistochemistry, treatment, and prognosis of two hospitalized patients and one consulted patient diagnosed with IGN were reviewed. In addition, one patient underwent molecular genetic analysis. This study conformed to the provisions of the institutional ethics committee and the Declaration of Helsinki (as revised in 2013). The patient’s parents shared all procedures, including treatment, and signed written informed consent. Written informed consent was obtained for the publication of any potentially identifiable images or data included in this article.

### Histopathology

Specimens for all patients were obtained from endoscopic biopsy or surgical resection, fixed in 10% buffered formalin, dehydrated in graded concentrations of ethyl alcohol, and embedded in paraffin. Then, they underwent routine staining for hematoxylin and eosin (H&E).

### Immunohistochemistry

Additional 4 μm sections were deparaffinized, rehydrated, and pretreated with 3% H_2_O_2_ to eliminate endogenous peroxidase activity. Moreover, they were treated with ethylenediaminetetraacetic acid (EDTA) (pH 9) or citrate buffer (pH 6) for heat-mediated antigen retrieval before commencing with the immunohistochemical (IHC) staining protocol. The primary antibodies used included S-100 (clone 4C4.9, ready-to-use solution), protein gene product 9.5 (PGP9.5) (polyclone, ready-to-use solution), synaptophysin (Syn) (clone MX038, ready-to-use solution), paired-like homeobox 2B (PHOX2B) (clone EP312, ready-to-use solution), neuron-specific nuclear protein (NeuN) (clone A60, ready-to-use solution), neurofilament (NF) (clone 2F11, ready-to-use solution), neuron-specific enolase (NSE) (clone 3-3-C, ready-to-use solution), SRY-related HMG-box 10 (SOX10) (clone EP268, ready-to-use solution), Vimentin (clone MX034, ready-to-use solution), Ki67 (clone MX006, ready-to-use solution), CD117 (clone YR145, ready-to-use solution), CD34 (clone QBEnd/10, ready-to-use solution), and CD163 (clone MX081, ready-to-use solution), which were purchased from http://www.maxim.com.cn (Fuzhou, China) and http://www.gzlbp.com (Guangzhou, China). The sections were incubated overnight at 4°C, followed by incubating with a general secondary antibody for 1 h at room temperature. Finally, the sections were developed with diaminobenzidine (DAB) and counterstained with hematoxylin. Omitting the first antibody was prepared as the negative control.

### Molecular Genetic Analysis

The EDTA-anticoagulated whole blood samples were collected from one patient and his parents. Trio-WES was performed by MyGenostics (a commercial genetic testing company) using the Illumina HiSeq X ten platform. DNA libraries were prepared with TruSeq DNA Library Preparation Kit following the manufacturer’s instructions. The raw reads were mapped to the reference human genome (GRCh37/hg19). Genome Analysis Toolkit (GATK)^1^ was applied for variation calling to summarize single nucleotide variants (SNVs) and indels. The ANNOVAR software and Enliven^®^ Variants Annotation Interpretation System were employed for annotation and interpretation. Data were filtered in 1,000 Genome,^2^ NHLBI Exome Sequencing Project (ESP6500),^3^ Genome Aggregation Database (gnomAD),^4^ dbSNP152,^5^ and Exome Aggregation Consortium (ExAC).^6^ Damage prediction of the genetic variants was conducted by Combined Annotation Dependent Depletion (CADD)^7^ for scoring and Mutation Significance Cutoff (MSC)^8^ for further comparing. The MSC server was applied to CADD, PolyPhen 2,^9^ and SIFT,^10^ with a 99% confidence interval and database source of HGMD and ClinVar.^11^ Genomics England PanelApp,^12^ a crowdsourcing tool, was utilized for analysis based on the variant-disease and gene-disease associations. Human Phenotype Ontology (HPO),^13^ Online Mendelian Inheritance in Man (OMIM),^14^ and HGMD database were used to match phenotype descriptions with variant and gene prioritization results. According to the American College of Medical Genetics and Genomics (ACMG) guidelines, genetic variants were classified as pathogenic, likely pathogenic, variants of uncertain significance (VUS), likely benign, and benign. The pathogenicity of filtered-out variants was predicted by MutationTaster^15^ as well. We conducted protein modeling of RTEL1 by SWISS-model^16^ with a UniProtKB code Q9NZ71, and the mutated structure was analyzed and visualized using PyMol.^17^ In addition, a total of 647 colorectal cancer samples and 51 normal controls from TCGA^18^ COADREAD were selected. The differential expression of *RTEL1* mRNA was analyzed by the Wilcoxon rank-sum test and visualized by ggplot2 (3.3.3 version). The protein expression of RTEL1 in Caco-2 cells was displayed by The Human Protein Atlas (HPA), using an antibody coded HPA078328.

## Results

### Patient 1

**FIGURE 1:**
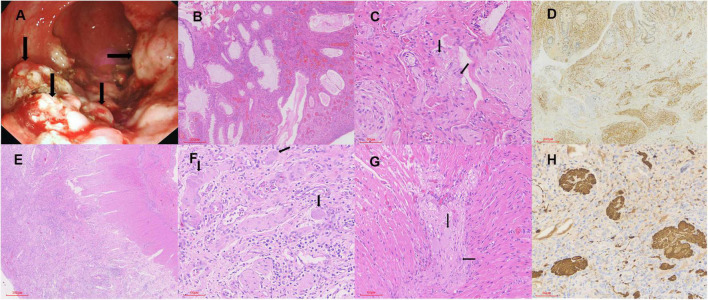
The endoscopic and pathological findings. **(A)** Multiple polyps (arrows) with paving stone-like changes in the descending colon. **(B)** Gland hyperplasia, cavity expansion, interstitial blood vessel hyperplasia, and neutrophil infiltration in the lesions (H&E × 40). **(C)** Nerve fibers, Schwann cells, and scattered ganglion cells (arrows) in the proliferated nerve plexus (H&E × 200). **(D)** The neoplastic hyperplasia is positive for S-100 (IHC × 40). **(E)** Significant inflammatory exudation and necrosis of the mucosal layer of the intestinal wall (H&E × 40). **(F,G)** The nerve plexus in the submucosa and myenteric is significantly proliferated and enlarged, with ganglion cells (arrows), nerve fibers, and Schwann matrix included (H&E × 200). **(H)** The neural elements are positive for PGP9.5 (IHC × 200).

A 6-year-old boy with anal masses and bloody stools for 2 years was admitted to our hospital. The patient was born to healthy non-consanguineous parents as the second child in the family, and the sibling was healthy. No family members had similar symptoms. Physical examination on admission showed normal growth and development, with a height of 122 cm and a weight of 25.5 kg. There was no tenderness and no palpable mass in the abdomen. By finger examination, many spherical masses with diameters of 0.2–0.5 cm were palpable in the rectum, protruding from the surface of the rectal mucosa. Except for a reduction in hemoglobin (101 g/L) and red blood cell count (3.96 × 10^12^/L), other routine blood test indicators were in the normal range. Liver and kidney function and coagulation parameters were unremarkable. Colonoscopy revealed multiple polyps with paving stone-like changes in the descending colon (Figure 1A) of 10 cm from the anal orifice, extending outward to the dentate line and partly located in the rectal fold. The polyps were removed by electrocoagulation and cauterization. Gland hyperplasia, cavity expansion, interstitial blood vessel hyperplasia, necrosis, neutrophil infiltration, and hemosiderin deposits were observed in the lesions under microscopy (Figure 1B). The preliminary impression was juvenile polyps. In addition, there was a proliferation of the nerve plexus in the lamina propria and submucosa, consisting of nerve fibers, Schwann cells, and scattered ganglion cells (Figure 1C). IHC staining showed that the neoplastic hyperplasia was positive for S-100 (Figure 1D), PGP9.5, and Syn, but negative for PHOX2B. The Ki67 index was 5% approximately. Based on the findings, a diagnosis of IGN (ganglioneuromatous polyposis subtype) was made. To investigate systemic disorders correlating with IGN, an additional examination was performed. Cutaneous cafe’-au-lait spots and neurofibromas, thyroid and adrenal lumps, or trichilemmomas and “cobblestone” tongue lesions were all absent, and serum calcitonin and urine catecholamine levels were normal. However, due to the unavailability of genetic testing in this patient, NF1, MEN2B, and Cowden syndrome cannot be ruled out. The mutations in *NF1*, *RET*, and *PTEN* are pending to be detected. During the 3-year follow-up, the patient had multiple recurrences but with no malignant transformation.

### Patient 2

A boy aged 1 year and 4 months with recurrent diarrhea for 2 months was referred to our hospital. Prior to admission, the patient had been hospitalized in the intensive care unit (ICU) of another hospital for 77 days due to severe pneumonia. After anti-infective therapy and invasive ventilator-assisted ventilation, the patient’s pneumonia improved. Subsequently, the patient repeatedly developed rashes and loose stools, which resembled a dilute water sample, more than 20 times a day. The boy was delivered by a full-term cesarean section and he weighed 4.1 kg at birth and was assessed as having macrosomia. The parents both claimed to be in good health, though his mother had a miscarriage in the third month of her first pregnancy for unknown reasons. No family members had similar symptoms. Compared with his peers, the patient’s growth and development were normal, with a height of 79.5 cm and a weight of 8.9 kg on physical examination at admission. Routine blood tests revealed elevated C-reactive protein (51.8 mg/L), neutrophil percentage (66.9%), and white blood cell count (8.4 × 10^9^/L), and decreased lymphocyte percentage (27.1%). Albumin (22.90 g/L), alanine aminotransferase (7.0 U/L), and lactate dehydrogenase (361 U/L) were at low levels. Routine stool examination displayed 3–5 white blood cells per high-power field (HPF), without pathogens. Metagenome sequencing screened out human cytomegalovirus (HHV-5) with high confidence. However, no nucleotide sequences of other pathogens were detected, such as *Clostridium difficile*. Abdominal computed tomography (CT) showed that the liver was slightly enlarged, with a reduced density. The rectum, sigmoid colon, and partial small intestine were dilated and effused, and the colon wall was slightly thickened. In addition, enlargement of multiple mesenteric lymph nodes and minimal ascites was noted. Although colonoscopy during the patient’s previous hospitalization showed erosive colitis with superficial ulcers and granulation tissue formation, our surgeon performed another colonoscopy to confirm the intestinal manifestation, when the colonoscope explored the transverse colon, a change to laparotomy was required due to difficulty in entering the region. In the surgical view, there was a moderate amount of clear ascites in the abdominal cavity. Starting from 15 cm of the flexor ligament, the small intestine exhibited multiple segmental stenoses and dilatations, with poor peristaltic function. The entire small intestinal mucosa presented pseudomembranous changes, raising suspicion of pseudomembranous enteritis. After clearing the necrotic mucosa, the entire thickness of the intestinal sample was taken and sent for pathological examination, followed by enterotomy. Histologically, significant inflammatory exudation and necrosis of the mucosal layer of the intestinal wall with ulcer formation, as well as eosinophils and histiocyte-like cells, were detected (Figure 1E). The nerve plexus in the submucosa and myenteria was significantly proliferated and enlarged, including ganglion cells, nerve fibers, and Schwann matrix (Figures 1F,G). Histiocyte-like cells in the necrotic region were immunoreactive for CD163, and neural elements were positive for Syn (Figure 1H) and PGP9.5. Hence, he was diagnosed with IGN (diffuse ganglioneuromatosis). The patient received symptomatic and supportive treatment of anti-infection and albumin supplementation. Because of severe and uncontrollable inflammation, the patient’s condition gradually deteriorated, and he unfortunately died 3 months after surgery.

### Patient 3

Case 3, a consultation case, involved an 8-year-old boy who had a history of bloody stools for 3 months. No family members had similar symptoms. The patient had been hospitalized in a local hospital. Routine stool examination revealed occult blood positivity, though routine blood examination and coagulation function were normal. Electronic colonoscopy revealed multiple colorectal polyps. Two sigmoid colon and rectal polyps were removed, with a pathological diagnosis of juvenile polyps. Subsequently, intestinal endoscopic submucosal dissection (ESD) was performed, and 17 sigmoid colon polyps were excised. Then, the patient’s pathological slides were transferred to our Department of Pathology for consultation. Histologically, there were many acute and chronic inflammatory cell infiltrations in the intestinal mucosa, and hyperplastic nerve plexuses were seen in the lamina propria and submucosa, in which several ganglion cells were surrounded by nerve fibers and Schwann cells. The cells were positive for Vimentin, NF, NSE, S-100, SOX10, and Syn but negative for NeuN, CD117, and CD34. Therefore, the result of the consultation was IGN (ganglioneuromatous polyposis subtype). Because it was a consultation case, no more clinical examination information was obtained. The patient’s parents denied any family history of MEN2B, NF1, or Cowden syndrome, and declined genetic testing to detect any germline mutations in *RET*, *NF1*, and *PTEN*. The patient’s subsequent follow-up indicated recurrence. During the 2-year follow-up, there were still more than ten broad-based polypoid lesions in the descending colon, sigmoid colon, and rectum, which all indicated IGN.

The main manifestations of the 3 patients are provided in Table 1.

**TABLE 1:** The main manifestations of the case series.

	Patient 1	Patient 2	Patient 3
Gender	Male	Male	Male
Age (years)	6	1.3	8
Bloody stools	Present	Absent	Present
Diarrhea	Absent	Present	Absent
Constipation	Absent	Absent	Absent
Growth and development	Normal	Normal	Normal
Systemic disorders	None	None	None
Family history	None	None	None
Disease location	Descending colon	Small intestine	Sigmoid colon and rectum
Mimicry	Juvenile polyps	Pseudomembranous enteritis	Juvenile polyps
Pathological subtype	Ganglioneuromatous polyposis	Diffuse ganglioneuromatosis	Ganglioneuromatous polyposis
Genetic findings by WES	Not available	*RTEL1* (c.296C > T/p.Pro99Leu), *F11* (c.1489C > T/p.Arg497Xaa), *NBAS* (c.1514delC/p.Pro505Hisfs*15), and *FECH* (c.315-48T > C/splicing)	Not available
Mutations in *RET*,*NF1*, and *PTEN*	Not available	Not detected	Not available
Treatment	Polypectomy	Surgical resection	Polypectomy
Outcome	Relapsed during 3-year follow-up	Died 3 months after surgery	Relapsed during 2-year follow-up

### Genetic Findings

**FIGURE 2:**
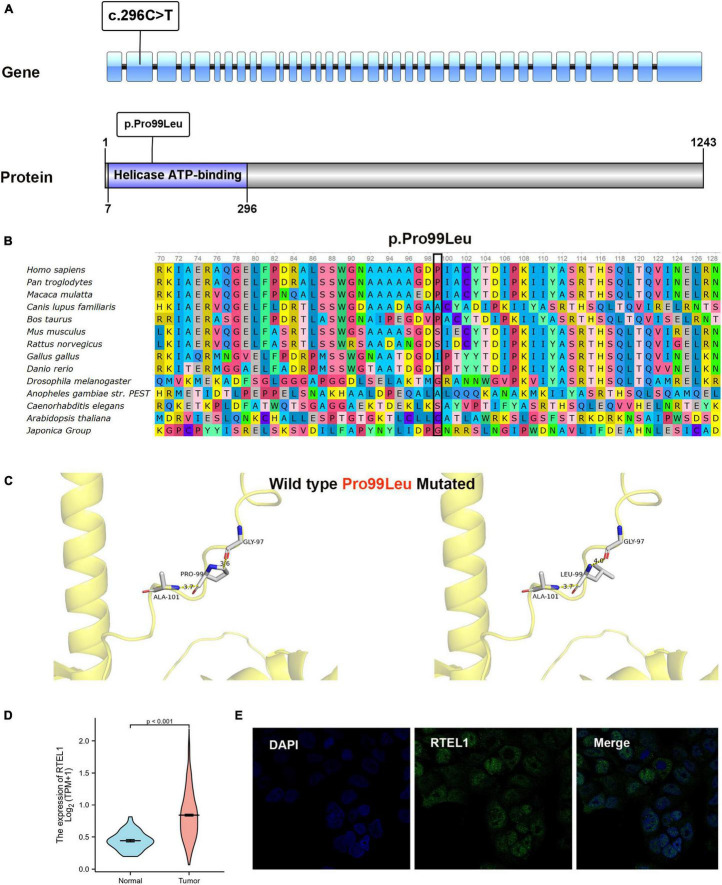
Bioinformatics analysis results of RTEL1. **(A)** The location of variant c.296C > T (p.Pro99Leu) in gene and protein. **(B)** Conservation status of amino acid residue of the mutation site across various species. **(C)** Wild and mutated type of the p.Pro99Leu variant compared by PyMol. **(D)** The differential expression of *RTEL1* mRNA in colorectal cancer (*n* = 647) and normal control (*n* = 51) in TCGA. **(E)** The protein expression of RTEL1 in colorectal adenocarcinoma cell line Caco2 in the Human Protein Atlas.

In Patient 2, a germline heterozygous variation, c.296C > T, was found in *RTEL1* with the transcript of NM_032957 by WES. The variant was inherited from the healthy father, leading to a change of proline to leucine at the amino acid position 99 (p.Pro99Leu) in the domain of helicase ATP-binding (Figure 2A), which was not reported in the HGMD. The amino acid residue of the base substitution (p.Pro99Leu) was not conserved across various species (Figure 2B). The CADD score of c.296C > T was 12.030, predicted as an uncertain impact by MSC. Comprehensively, c.296C > T (absent from controls PM2 + insufficient evidence *in silico* BP4) was evaluated to be VUS according to the guideline of the ACMG, which need further study to confirm the association between the variant and phenotype. The nomenclature of variants was based on the recommendations of the Human Genome Variation Society (HGVS).^19^ Wild-type and mutated RTEL1 were modeled by PyMol (see text footnote 17)that showed no effect of Pro99Leu residue change on polar contact with the amino acids Gly97 and Ala101 (Figure 2C). In the TCGA database, the differential expression of *RTEL1* mRNA in colorectal cancer was higher than normal control with statistical significance (*p* < 0.001) (Figure 2D). The protein expression of RTEL1 in Caco-2 cells was exhibited by data from HPA (Figure 2E). There were another 3 pathogenic or likely pathogenic variants identified. The heterozygous variants in *F11* (c.1489C > T/p.Arg497Xaa) and *NBAS* (c.1514delC/p.Pro505Hisfs*15) were inherited from the healthy mother, while the *FECH* (c.315-48T > C/splicing) was inherited from father and mother. The genetic variants with potential significance are presented in Table 2.

**TABLE 2:** Potential pathogenic genetic variants detected in Patient 2.

Gene symbol	Mutation site	Zygosity	ACMG pathogenicity	Selected diseases associated with the gene	Source of variation
*RTEL1*	c.296C > T/p.Pro99Leu	Heterozygous	Uncertain	Hoyerall-Hreidarsson syndrome, dyskeratosis congenita	Father
*F11*	c.1489C > T/p.Arg497Xaa	Heterozygous	Pathogenic	Factor XI deficiency	Mother
*NBAS*	c.1514delC/p.Pro505Hisfs*15	Heterozygous	Likely pathogenic	Short stature, optic nerve atrophy, and Pelger-Huet anomaly	Mother
*FECH*	c.315-48T > C/splicing	Heterozygous	Pathogenic	Erythropoietic protoporphyria	Father and Mother

## Discussion

The IGN occurs in both children and adults, mostly in children younger than 15 years. The pediatric patients aged from 2 days to 14 years, with a median age of 6 years, and with a slightly higher incidence in males. The most common location is the colorectum, followed by the terminal ileum and the entire intestine. The stomach or pancreatic head may be involved simultaneously (11, 12), and a few occurrences in the gallbladder or bladder have been reported (13, 14). Usually, patients present with abdominal pain, diarrhea, vomiting, bloody stools, constipation, and obstruction, which in some cases is accompanied by iron-deficiency anemia, elevated serum vasoactive intestinal peptide and hypokalemia, and growth and motor development retardation (15–20). Many patients have systemic disorders, predominantly MEN2B, followed by Cowden syndrome and NF1, presenting thyroid and adrenal tumors, trichilemmomas and “cobblestone” tongue lesions, or cutaneous cafe’-au-lait spots and neurofibromas (4–6, 16, 17, 21–24). Endoscopically, pedunculated or sessile polyps are common, ranging from single to numerous, with a diameter of 0.1–17 cm, and may be misdiagnosed as juvenile polyps or GIST (8, 17). Non-polyposis lesions manifest as intestinal stenosis or inflammatory lesions of mucosal ulcers, leading to misdiagnosis with Hirschsprung’s disease or Crohn’s disease (7, 9, 16, 20, 21). The age, sex, and clinical and endoscopic findings of our case series are consistent with those reported previously, but there were no signs of systemic disorders, which need further surveillance and validation. Patient 2 presented with severe lung and intestinal inflammation, and notable intestinal mucosal necrosis mimicked pseudomembranous enteritis, which is not reported previously. With no positive findings based on multiple mucosal biopsies, a definitive diagnosis was delayed, illustrating the importance of full-thickness biopsy.

Histologically, IGN is defined by the abnormal proliferation of ganglion cells, nerve fibers, and Schwann cells in the enteric nervous system. IGN can be divided into polypoid GN, ganglioneuromatous polyposis, and diffuse ganglioneuromatosis (3). Polypoid ganglioneuroma is a small, single polypoid lesion ≤ 2 cm in size composed of hyperplastic Schwann cells and scattered ganglion cells involving the mucosa and submucosa (17). Ganglioneuromatous polyposis presents as multiple intestinal polyps, often > 20 in quantity and ≤ 2.2 cm in diameter, endoscopically similar to familial adenomatous polyposis (FAP) or juvenile polyposis, which may lead to misdiagnosis (23). The tumor cells of diffuse ganglioneuromatosis are nodular or diffusely proliferating, and they invade the intestinal wall and may be accompanied by the proliferation of the submucosa, myenteric nerve plexus, or even mesenteric nerve plexus (4, 6–8, 24–26). On IHC staining, Schwann cells express CD56, SOX10, and S-100, and ganglion cells express NSE, CD56, Syn, Calretinin, NeuN, and S-100 (6, 8, 12, 19, 20, 25–27). According to the above criteria, 2 of our cases showed multiple colorectal polyps (≥ 20), and neurofibril and ganglion cell hyperplasia was detected in the juvenile polyps under the microscope; thus, they were diagnosed as ganglioneuromatous polyposis. Another case showed significant pseudomembranous inflammation and stenosis changes in the entire small intestine wall. In addition to inflammation and necrosis by microscopy, the nerve plexus in the submucosa and myenteria was significantly proliferated and enlarged, with ganglion cells, nerve fibers, and Schwann matrix, in line with the diagnosis of diffuse ganglioneuromatosis. IHC staining showed that the neoplastic hyperplasia was positive for S-100, PGP9.5, and Syn, which further supported the diagnosis. However, due to the uncommon nature of IGN and the possibility of coexistence with juvenile polyps, neurofibromas, or schwannoma, it is still challenging to reach timely and correct judgment in daily work.

Due to close relationship between IGN and hereditary tumor syndromes, diagnosis of IGN usually triggers genetic testing. *RET*, *PTEN*, or *NF1* mutations were detected to screen for MEN2B, Cowden syndrome, or NF1, respectively. Animal model studies have found that *RET*-activated mutations and *PTEN* deletion mutations can generate GN in mice through the PI3K/PTEN-AKT-S6K signaling pathway (28, 29). Whether the pathogenesis of IGN is consistent with the mechanism remains to be confirmed by more experiments. In our series, genetic testing for *RET*, *PTEN*, or *NF1* was not available in Patient 1 and Patient 3, and further work is required to rule out systemic disorders. Patient 2 underwent WES to investigate possible genetic syndromes, and a heterozygous *RTEL1* mutation (c.296C > T/p.Pro99Leu) was detected rather than a mutation in *RET*, *PTEN*, or *NF1*. Therefore, MEN2B, Cowden syndrome, and NF1 were excluded. *RTEL1* (regulator of telomere elongation helicase 1) (NM_032957, ENST00000508582) is located on chromosome 20 (chr20: 63,658,312-63,696,245), containing 35 exons encoding a protein of 1,243 amino acids, which functions in the stability, protection, and elongation of telomeres and interacts with proteins in the shelterin complex known to protect telomeres during DNA replication (30). *RTEL1* mutations have been associated with dyskeratosis congenita and Hoyeraal–Hreidarsson syndrome (31, 32). Ziv et al. (33) found that C.3791G > A in *RTEL1* was linked to infantile-onset ulcerative colitis and severe immunodeficiency, which is likely the result of aberrant telomere function in both immune and epithelial cells. Patient 2 harbored the novel c.296C > T in *RTEL1*, resulting in the change of proline to leucine at the amino acid position 99 (p.Pro99Leu) in the domain of helicase ATP-binding. Whether the mutation led to unstable telomere maintenance and drove the occurrence of IGN in patient 2 needs further analysis.

Additional mutations in *F11*, *NBAS*, and *FECH* were screened out in Patient 2, predicted as pathogenic or likely pathogenic. *F11* (coagulation factor XI) encodes coagulation factor XI of the blood coagulation cascade. The variant of c.1489C > T/p.Arg497Xaa has been found in factor XI deficiency (34). *NBAS* (NB-amplified sequence) encodes a protein with two leucine zipper domains, a ribosomal protein S14 signature domain and a Sec39-like domain. The protein is thought to be involved in Golgi-to-ER transport. Mutations in this gene are associated with short stature, optic nerve atrophy, and Pelger-Huet anomaly (35). The frameshift mutation (c.1514delC/p.Pro505Hisfs*15) in Patient 2 caused the amino acid at position 505 to be shifted 15 positions back and then terminated early, which is unique according to references. The protein encoded by *FECH* (ferrochelatase) is localized to the mitochondrion, where it catalyzes the insertion of the ferrous form of iron into protoporphyrin IX in the heme synthesis pathway. The c.315-48T > C/splicing has been reported to be associated with erythropoietic protoporphyria (36). However, the variants are not highly correlated with the phenotype of Patient 2 and the evidence for pathogenicity was insufficient.

Patients with IGN are usually treated with surgery. For polyp lesions, polypectomy alone is sufficient. If the lesions are too large or the intestinal stenosis is obvious, bowel resection should be performed. Although IGN is a benign tumor, many patients experience recurrence. There are 2 reported cases of IGN with concomitant adenocarcinoma or adenocarcinoma development at 12 years after diagnosis (37, 38). The significant differential expression of *RTEL1* in colorectal cancer and normal control in TCGA indicates that *RTEL1* may play a role in the progression of malignancy, while its contribution to IGN or adenocarcinoma that developed from IGN is unclear and worth investigating. Our case series involved polypectomy and surgical resection. After 3 months to 3 years follow-up, 2 cases of ganglioneuromatous polyposis recurred repeatedly, despite multiple polypectomy procedures. Intestinal resection should be considered in the future. Unfortunately, the excessive inflammatory response in another case of diffuse ganglioneuromatosis was not corrected, resulting in multiple organ dysfunction and death. These results demonstrate that seemingly benign IGN has a poor disease course, which should arouse the attention of clinicians.

## Conclusion

As a rare condition with enteric involvement, our case series expands the phenotypic and genotypic spectrum of pediatric IGN by mimicking pseudomembranous enteritis and carrying a novel germline mutation in *RTEL1* along with additional variants. The patients experience relapses or death. The unclear pathogenesis and unfavorable prognosis require more awareness and research.

## Data Availability Statement

The datasets presented in this article are not readily available because of patient privacy concerns. Requests to access the datasets should be directed to LC, doctchenlian@163.com.

## Ethics Statement

The studies involving human participants were reviewed and approved by the Children’s Hospital, Fudan University and Anhui Provincial Children’s Hospital. Written informed consent to participate in this study was provided by the participants’ legal guardian/next of kin.

## Author Contributions

YF and YZ drafted and revised the manuscript. RD and Y-ZW collected and analyzed the data. LC and GC designed the work and approved the final version to be published. All authors contributed to the article and approved the submitted version.

## Conflict of Interest

The authors declare that the research was conducted in the absence of any commercial or financial relationships that could be construed as a potential conflict of interest.

## Publisher’s Note

All claims expressed in this article are solely those of the authors and do not necessarily represent those of their affiliated organizations, or those of the publisher, the editors and the reviewers. Any product that may be evaluated in this article, or claim that may be made by its manufacturer, is not guaranteed or endorsed by the publisher.

## Footnotes

1
https://gatk.broadinstitute.org

2
www.1000genomes.org

3
https://esp.gs.washington.edu

4
https://gnomad.broadinstitute.org

5
https://www.ncbi.nlm.nih.gov/snp

6
gs://gnomad-public/legacy

7
https://cadd.gs.washington.edu

8
https://lab.rockefeller.edu/casanova/MSC

9
http://genetics.bwh.harvard.edu/pph2

10
https://sift.bii.a-star.edu.sg/www/SIFT_indels2.html

11
https://www.ncbi.nlm.nih.gov/clinvar

12
https://panelapp.genomicsengland.co.uk

13
https://hpo.jax.org

14
https://www.omim.org

15
http://www.mutationtaster.org

16
https://www.swissmodel.expasy.org

17
http://www.pymol.org

18
https://portal.gdc.cancer.gov/

19
http://www.hgvs.org/varnomen

## References

[1] Cohn SL Pearson AD London WB Monclair T Ambros PF Brodeur GM The international neuroblastoma risk group (INRG) classification system: an INRG Task Force report. *J Clin Oncol.* (2009) 27:289–97. 10.1200/JCO.2008.16.6785 19047291 PMC2650388

[2] Schulte JH Eggert A. Neuroblastoma. *Crit Rev Oncog.* (2015) 20:245–70. 10.1615/critrevoncog.2015014033 26349419

[3] Shekitka KM Sobin LH. Ganglioneuromas of the gastrointestinal tract. Relation to von recklinghausen disease and other multiple tumor syndromes. *Am J Surg Pathol.* (1994) 18:250–7. 7906923

[4] Herranz Bachiller MT Barrio Andres J Pons F Alcaide Suarez N Ruiz-Zorrilla R Sancho Del Val L Diffuse intestinal ganglioneuromatosis an uncommon manifestation of Cowden syndrome. *World J Gastrointest Oncol.* (2013) 5:34–7. 10.4251/wjgo.v5.i2.34 23556055 PMC3613769

[5] Gfroerer S Theilen TM Fiegel H Harter PN Mittelbronn M Rolle U. Identification of intestinal ganglioneuromatosis leads to early diagnosis of MEN2B: role of rectal biopsy. *J Pediatr Surg.* (2017) 52:1161–5. 10.1016/j.jpedsurg.2016.10.054 27899172

[6] Iwamuro M Omote R Tanaka T Sunada N Nada T Kondo Y Diffuse intestinal ganglioneuromatosis showing multiple large bowel ulcers in a patient with neurofibromatosis Type 1. *Intern Med.* (2017) 56:3287–91. 10.2169/internalmedicine.8671-16 29021449 PMC5790715

[7] Charagundla SR Levine MS Torigian DA Campbell MS Furth EE Rombeau J. Diffuse intestinal ganglioneuromatosis mimicking Crohn’s disease. *AJR Am J Roentgenol.* (2004) 182:1166–8. 10.2214/ajr.182.5.1821166 15100112

[8] Yin X Chen X Shu R Shen C Yin Y Cai Z Diffuse ileal ganglioneuromatosis mimicking a gastrointestinal stromal tumor: a case report. *Medicine (Baltimore).* (2019) 98:e16305. 10.1097/MD.0000000000016305 31277169 PMC6635301

[9] Mohammed Kheir H Dayoub M Haidar N Mansour H Omran A Ibrahim A incidental delayed diagnosis of isolated diffuse ganglioneuromatosis caused the death of a 6-year-old boy: case report. *Clin Med Insights Case Rep.* (2021) 14:11795476211049864. 10.1177/11795476211049864 34629921 PMC8493302

[10] Riley DS Barber MS Kienle GS Aronson JK von Schoen-Angerer T Tugwell P CARE guidelines for case reports: explanation and elaboration document. *J Clin Epidemiol.* (2017) 89:218–35. 10.1016/j.jclinepi.2017.04.026 28529185

[11] Shulman DI McClenathan DT Harmel RP Qualman SJ O’Dorisio TM. Ganglioneuromatosis involving the small intestine and pancreas of a child and causing hypersecretion of vasoactive intestinal polypeptide. *J Pediatr Gastroenterol Nutr.* (1996) 22:212–8. 10.1097/00005176-199602000-00015 8642497

[12] Atluri DK Ganesan S Ferguson RD. Education and imaging. Gastrointestinal: intestinal ganglioneuromatosis. *J Gastroenterol Hepatol.* (2008) 23:160. 10.1111/j.1440-1746.2007.05254.x 18171355

[13] Scheithauer BW Santi M Richter ER Belman B Rushing EJ. Diffuse ganglioneuromatosis and plexiform neurofibroma of the urinary bladder: report of a pediatric example and literature review. *Hum Pathol.* (2008) 39:1708–12. 10.1016/j.humpath.2008.02.019 18656232

[14] Sakuma T Hirota M Ohashi H Kakudo K Kawano K. Extensive ganglioneuromatosis of gallbladder. *Int J Surg Pathol.* (2011) 19:524–6. 10.1177/1066896908324129 18805869

[15] Rescorla FJ Vane DW Fitzgerald JF West KW Grosfeld JL. Vasoactive intestinal polypeptide-secreting ganglioneuromatosis affecting the entire colon and rectum. *J Pediatr Surg.* (1988) 23:635–7. 10.1016/s0022-3468(88)80634-1 2849648

[16] Smith VV Eng C Milla PJ. Intestinal ganglioneuromatosis and multiple endocrine neoplasia type 2B: implications for treatment. *Gut.* (1999) 45:143–6. 10.1136/gut.45.1.143 10369718 PMC1727575

[17] Nguyen AT Zacharin MR Smith M Hardikar W. Isolated intestinal ganglioneuromatosis with a new mutation of RET proto-oncogene. *Eur J Gastroenterol Hepatol.* (2006) 18:803–5. 10.1097/01.meg.0000224473.66675.ad 16772843

[18] Moon SB Park KW Jung SE Lee SC. Vasoactive intestinal polypeptide-producing ganglioneuromatosis involving the entire colon and rectum. *J Pediatr Surg.* (2009) 44:e19–21. 10.1016/j.jpedsurg.2008.11.051 19302839

[19] Fernandes A Ferreira AM Serra P Carvalho L. Intestinal ganglioneuromatosis: an unusual aetiology for occult gastrointestinal bleeding. *BMJ Case Rep.* (2015) 2015:bcr2015211764. 10.1136/bcr-2015-211764 26424825 PMC4593261

[20] Gizard E Kalouche I Merlin P Peyrin-Biroulet L. Ileal ulcers not responding to infliximab therapy: think about intestinal ganglioneuromatosis. *Dig Liver Dis.* (2015) 47:257–8. 10.1016/j.dld.2014.11.001 25499657

[21] Anderson TE Spackman TJ Schwartz SS. Roentgen findings in intestinal ganglioneuromatosis. Its association with medullary thyroid carcinoma and pheochromocytoma. *Radiology.* (1971) 101:93–6. 10.1148/101.1.93 5111989

[22] DeSchryver-Kecskemeti K Clouse RE Goldstein MN Gersell D O’Neal L. Intestinal ganglioneuromatosis. A manifestation of overproduction of nerve growth factor? *N Engl J Med.* (1983) 308:635–9. 10.1056/NEJM198303173081106 6131380

[23] Vinitsky A Zaleski CA Sajjad SM McPherson EW. Intestinal ganglioneuromatosis: unusual presentation of Cowden syndrome resulting in delayed diagnosis. *Am J Med Genet A.* (2013) 161A:1085–90. 10.1002/ajmg.a.35731 23512313

[24] Rosenfeld EH Chumpitazi BP Castro E Naik-Mathuria B. Diffuse intestinal ganglioneuromatosis causing severe intestinal dysmotility in a child with a PTEN Mutation. *J Pediatr Gastroenterol Nutr.* (2019) 68:e35–7. 10.1097/MPG.0000000000002072 29927861

[25] Matthews MA Adler BH Arnold MA Kumar S Carvalho R Besner GE. Diffuse intestinal ganglioneuromatosis in a child. *J Pediatr Surg.* (2013) 48:1129–33. 10.1016/j.jpedsurg.2013.03.066 23701793 PMC4076949

[26] Lu C Qiu Y Lu X Li G Bu H. Synchronous diffuse ganglioneuromatosis and multiple schwannomas of the colon: a case report and literature review. *Exp Ther Med.* (2015) 9:733–6. 10.3892/etm.2015.2212 25667620 PMC4316863

[27] Mauro A Zenzeri L Esposito F Gaglione G Strisciuglio C Pilozzi E Isolated intestinal ganglioneuromatosis: case report and literature review. *Ital J Pediatr.* (2021) 47:80. 10.1186/s13052-021-01024-5 33785023 PMC8008650

[28] Sweetser DA Froelick GJ Matsumoto AM Kafer KE Marck B Palmiter RD Ganglioneuromas and renal anomalies are induced by activated RET(MEN2B) in transgenic mice. *Oncogene.* (1999) 18:877–86. 10.1038/sj.onc.1202376 10023663

[29] Puig I Champeval D De Santa Barbara P Jaubert F Lyonnet S Larue L. Deletion of PTEN in the mouse enteric nervous system induces ganglioneuromatosis and mimics intestinal pseudoobstruction. *J Clin Invest.* (2009) 119:3586–96. 10.1172/JCI39929 19884655 PMC2786803

[30] Vannier JB Sarek G Boulton SJ. RTEL1: functions of a disease-associated helicase. *Trends Cell Biol.* (2014) 24:416–25. 10.1016/j.tcb.2014.01.004 24582487

[31] Ballew BJ Yeager M Jacobs K Giri N Boland J Burdett L Germline mutations of regulator of telomere elongation helicase 1, RTEL1, in Dyskeratosis congenita. *Hum Genet.* (2013) 132:473–80. 10.1007/s00439-013-1265-8 23329068 PMC3600110

[32] Le Guen T Jullien L Touzot F Schertzer M Gaillard L Perderiset M Human RTEL1 deficiency causes Hoyeraal-Hreidarsson syndrome with short telomeres and genome instability. *Hum Mol Genet.* (2013) 22:3239–49. 10.1093/hmg/ddt178 23591994

[33] Ziv A Werner L Konnikova L Awad A Jeske T Hastreiter M An RTEL1 mutation links to infantile-onset ulcerative colitis and severe immunodeficiency. *J Clin Immunol.* (2020) 40:1010–9. 10.1007/s10875-020-00829-z 32710398

[34] Mitchell M Mountford R Butler R Alhaq A Dai L Savidge G Spectrum of factor XI (F11) mutations in the UK population–116 index cases and 140 mutations. *Hum Mutat.* (2006) 27:829. 10.1002/humu.9439 16835901

[35] Maksimova N Hara K Nikolaeva I Chun-Feng T Usui T Takagi M Neuroblastoma amplified sequence gene is associated with a novel short stature syndrome characterised by optic nerve atrophy and Pelger-Huet anomaly. *J Med Genet.* (2010) 47:538–48. 10.1136/jmg.2009.074815 20577004 PMC2921285

[36] Gouya L Martin-Schmitt C Robreau AM Austerlitz F Da Silva V Brun P Contribution of a common single-nucleotide polymorphism to the genetic predisposition for erythropoietic protoporphyria. *Am J Hum Genet.* (2006) 78:2–14. 10.1086/498620 16385445 PMC1380220

[37] Qiao S Iwashita T Ichihara M Murakumo Y Yamaguchi A Isogai M Increased expression of glial cell line-derived neurotrophic factor and neurturin in a case of colon adenocarcinoma associated with diffuse ganglioneuromatosis. *Clin Neuropathol.* (2009) 28:105–12. 10.5414/npp28105 19353842

[38] Oh JS Hong SW Noh JH Yoon J Kang HJ Park YS Development of colon cancer in a patient with longstanding colonic diffuse ganglioneuromatosis: a case report. *Clin Endosc.* (2021). 10.5946/ce.2021.013 [Epub ahead of print]. 33657783 PMC9178138

