# Supplementary material for: A Case Series of Pediatric Intestinal Ganglioneuromatosis With Novel Phenotypic and Genotypic Profile
Source: Front Med (Lausanne). 2022 Jun 16;9:883958. doi: 10.3389/fmed.2022.883958 (PMC9243541; doi:10.3389/fmed.2022.883958)
Supplement: Supplementary file 1 [file Data_Sheet_1.pdf]

CARE case report guidelines

CARE Checklist of information to include when writing a case report

(cc) BY-NC-ND

Topic	Item	Checklist item description	Reported on Line
Title	1	The diagnosis or intervention of primary focus followed by the words "case report"	Line 1 to 2
Key Words	2	2 to 5 key words that identify diagnoses or interventions in this case report, including "case report"	Line 17 to 18
Abstract (no references)	3a	Introduction: What is unique about this case and what does it add to the scientific literature?	Line 4 to 6
	3b	Main symptoms and/or important clinical findings	Line 8 to 12
	3c	The main diagnoses, therapeutic interventions, and outcomes	Line 12 to 14
	3d	Conclusion—What is the main "take-away" lesson(s) from this case?	Line 14 to 16
Introduction	4	One or two paragraphs summarizing why this case is unique (**may include references**)	Line 20 to 49
Patient Information	5a	De-identified patient specific information.	Line 126, 156, 201
	5b	Primary concerns and symptoms of the patient.	Line 126, 156, 201
	5c	Medical, family, and psycho-social history including relevant genetic information	Line 125 to 245
	5d	Relevant past interventions with outcomes	Line 125 to 245
Clinical Findings	6	Describe significant physical examination (PE) and important clinical findings.	Line 125 to 245
Timeline	7	Historical and current information from this episode of care organized as a timeline	Line 125 to 245
Diagnostic Assessment	8a	Diagnostic testing (such as PE, laboratory testing, imaging, surveys).	Line 122 to 237
	8b	Diagnostic challenges (such as access to testing, financial, or cultural)	Line 151 to 152, 217 to 218
	8c	Diagnosis (including other diagnoses considered)	Line 147, 195, 215
	8d	Prognosis (such as staging in oncology) where applicable	Line 154, 198, 219
Therapeutic Intervention	9a	Types of therapeutic intervention (such as pharmacologic, surgical, preventive, self-care)	Line 125 to 245
	9b	Administration of therapeutic intervention (such as dosage, strength, duration)	Line 125 to 245
	9c	Changes in therapeutic intervention (with rationale)	Line 125 to 245
Follow-up and Outcomes	10a	Clinician and patient-assessed outcomes (if available)	Line 154, 198, 219
	10b	Important follow-up diagnostic and other test results	Line 154, 198, 219
	10c	Intervention adherence and tolerability (How was this assessed?)	Line 154, 198, 219
	10d	Adverse and unanticipated events	Line 154, 198, 219
Discussion	11a	A scientific discussion of the strengths AND limitations associated with this case report	Line 269 to 271, 311 to 313
	11b	Discussion of the relevant medical literature **with references**.	Line 247 to 361
	11c	The scientific rationale for any conclusions (including assessment of possible causes)	Line 363 to 367
	11d	The primary "take-away" lessons of this case report (without references) in a one paragraph conclusion	Line 363 to 367
Patient Perspective	12	The patient should share their perspective in one to two paragraphs on the treatment(s) they received	Line 58 to 59
Informed Consent	13	Did the patient give informed consent? Please provide if requested	**Yes** ☑ **No** ☐
